# Demystifying the mechanistic and functional aspects of p21 gene activation with double-stranded RNAs in human cancer cells

**DOI:** 10.1186/s13046-016-0423-y

**Published:** 2016-09-17

**Authors:** Huan-Lei Wu, Sen-Mao Li, Jia Hu, Xiao Yu, Hua Xu, Zhong Chen, Zhang-Qun Ye

**Affiliations:** 1 Department of Geriatrics, Tongji Hospital, Tongji Medical College, Huazhong University of Science and Technology, Wuhan, 430030 People’s Republic of China; 2 Department of Urology, Tongji Hospital, Tongji Medical College, Huazhong University of Science and Technology, Liberalization Avenue, No. 1095, Wuhan, 430030 People’s Republic of China; 3 Institute of Urology, Tongji Hospital, Tongji Medical College, Huazhong University of Science and Technology, Wuhan, 430030 People’s Republic of China

**Keywords:** saRNA, RNAa, p21, Promoter-targeted, Functional features

## Abstract

**Background:**

The recently identified phenomenon of double-stranded RNA (dsRNA)-mediated gene activation (RNAa) has been studied extensively, as it is present in humans, mice, and *Caenorhabditis elegans*, suggesting that dsRNA-mediated RNAa is an evolutionarily conserved mechanism. Previous studies have shown that dsP21-322 can induce tumor suppressor gene p21 expression in several human cancer cells. Nonetheless, the role of dsRNAs in the activation of gene expression, including their target molecules and associated key factors, remains poorly understood.

**Methods:**

Oligonucleotides were used to overexpress dsRNAs and dsControl. Real-time PCR and Western blotting were used to detect corresponding mRNA and protein expression, respectively. Fluorescence microscopy was used to examine the kinetics of dsRNA subcellular distribution. Luciferase reporter assays were performed to verify dsRNA target molecules. Chromatin immunoprecipitation (ChIP) assays were carried out to determine whether histone modification and other associated key factors are involved in saRNA-mediated p21 expression.

**Results:**

We demonstrated that dsRNA-mediated p21 induction in human cell lines is a common phenomenon. This process occurs at the transcriptional level, and the complementary p21 promoter is the intended dsRNA target. Additionally, ChIP assays indicated that p21 activation was accompanied by an increased enrichment of AGO1 and the trimethylation of histone H3K4 at dsRNA-targeted genomic sites.

**Conclusion:**

These data systematically reveal the mechanistic and functional aspects of ncRNA-mediated p21 activation in human cancer cells, which may be a useful tool to analyze gene function and aid in the development of novel drug targets for cancer therapeutics.

**Electronic supplementary material:**

The online version of this article (doi:10.1186/s13046-016-0423-y) contains supplementary material, which is available to authorized users.

## Background

In recent years, the regulation of gene expression by non-coding RNAs (ncRNAs) has become recognized as an evolutionarily conserved mechanism in biology. The well-studied smaller ncRNAs, such as short interfering RNAs (siRNAs) and microRNAs, can posttranscriptionally and epigenetically silence gene expression [1–4]. Many long ncRNAs, such as Xist and Air, have also been studied extensively in gene silencing, revealing epigenetic mechanisms [5, 6]. RNA interference (RNAi)-based gene silencing approaches have been performed in humans, and ongoing clinical trials show promise for treating cancer or providing alternatives to traditional small molecule therapies [7, 8].

Similar to double-stranded RNAs (dsRNAs), ncRNAs have more recently also been shown to induce gene expression by a phenomenon referred to as saRNA activation (RNAa) [9–18]; these types of dsRNAs are referred to as small activating RNAs (saRNAs). Several models of RNAa have been described, including the activation of transcription through complementary binding to target sequences within gene promoters and/or overlapping noncoding transcripts [19–23]. RNAa also offers an approach for gene regulation and represents a new strategy to stimulate gene expression. Although much is known regarding the mechanisms of gene silencing by small dsRNAs in human cells, little is known about the mechanism of RNA-induced gene activation.

p21^Waf1/Cip1^ (p21) [CDKN1A (cyclin-dependent kinase inhibitor 1A)] is a well-characterized cyclin-dependent kinase inhibitor that belongs to the Cip/Kip family of cyclin-dependent kinase inhibitors and is a key mediator of several cellular processes including cell death, DNA repair, senescence and aging [24, 25]. Transcriptional regulation is mediated by multiple transcription factors (p53, Sp1/Sp3 and c-Myc) and plays a critical role in the expression and activity of p21 [26]. p21 is a potent tumor suppressor gene (TSG) in cancer cells, although loss-of-function mutations in p21 are generally a rare occurrence [27]. Therefore, p21 is an ideal target for RNAa-mediated inhibition of tumor cell growth.

Previous studies have shown that a small activating RNA could markedly induce p21 expression by targeting the p21 gene promoter region in cancer cells, resulting in cell-cycle arrest and apoptosis in vitro [10, 28]. Recent observations also demonstrated that the intravesical delivery of saRNAs inhibited orthotopic bladder or xenograft prostate tumor growth by inducing p21 expression [29, 30]. Our group further demonstrated that saRNAs associated specifically with their intended target on the p21 promoter and interacted with the heterogeneous nuclear ribonucleoproteins A2/B1(hnRNPA2/B1) to mediate p21 induction [19]. However, the limited anecdotal evidence that is currently available makes it difficult to understand the molecular mechanisms of RNAa. In this study, we performed a detailed RNAa analysis using saRNAs and examined the kinetics of the saRNA subcellular distribution and the rate of p21 gene induction. We also showed that the upregulation of p21 expression by saRNAs occurs at the transcriptional level and is associated with activating epigenetic markers and argonaute 1 (AGO1) binding to saRNA-targeted genomic sites. These findings reveal functional and mechanistic aspects of RNAa that will help inform in vivo applications in medicine and aid in the development of RNAa as a laboratory tool.

## Methods

### dsRNA design and synthesis

A 21-nucleotide dsRNA targeting the p21 promoter at sequence position −322 relative to the transcriptional start site (dsP21-322) was used to activate p21 expression. A 21-nt dsRNA lacking significant homology to all known human sequences (dsControl) was used as a nonspecific control. The sequences of these dsRNAs are as follows: dsP21-322-S, 5’-CCA ACU CAU UCU CCA AGU A[dT][dT]-3’; dsP21-322-AS, 5’-UAC UUG GAG AAU GAG UUG G[dT][dT]-3’; dsControl-S, 5’-ACU ACU GAG UGA CAG UAG A[dT][dT]-3’; and dsControl-AS, 5’-UCU ACU GUC ACU CAG UAG U[dT][dT]-3’. Synthetic dsRNAs (including fluorescently labeled dsRNAs) were manufactured by Ribobio Inc. (Guangzhou, China). Human p53 siRNAs were purchased from Santa Cruz Biotechnology (sc-29435; Santa Cruz).

### Plasmids and antibodies

The dsP21-322 target site in the p21 promoter constructs (-395/-197) was PCR amplified using genomic DNA and verified by sequencing. The PCR fragment was cloned into the pGL3-Basic luciferase reporter vector upstream of the firefly luciferase gene to generate the pGL3-promoter p21 construct. Oligonucleotide sequences are listed in Additional file 1: Table S1. The following antibodies were used: monoclonal anti-AGO2 [ab57113, chromatin immunoprecipitation (ChIP) grade, for IP; Abcam]; monoclonal anti-AGO1(2A7) [015–22411, chromatin immunoprecipitation (ChIP) grade, for IP; Wako]; monoclonal anti-biotin (sc-53179, for IP; Santa Cruz); normal mouse IgG (12–371, for IP; Millipore); monoclonal anti-RNA polymerase II (05–623, for IP; Millipore); polyclonal anti-trimethyl-histone H3 (Lys4) (07–473, for IP; Millipore); polyclonal anti-p21(ab7960, for WB; Abcam); monoclonal anti-p53(DO-1) (sc-126, for WB; Santa Cruz); and monoclonal anti-GAPDH (ab9484, for WB; Abcam).

### Cell culture and transfection

All of the cell lines were from ATCC. PC-3, U2-OS, HeLa, 293T, SaOS2 and 5637 cell lines were maintained in RPMI medium 1640, and T-24, ACHN, NCI-H1299 and Hep3B cell lines were cultured in Dulbecco’s Modified Eagle’s Medium that was supplemented with 10 % FBS, 2 mM L-glutamine, penicillin (100 units/ml), and streptomycin (100 μg/ml) in a humidified atmosphere of 5 % CO_2_ maintained at 37 °C. MDA-MB-157 was cultured in L-15 media at 37 °C without CO_2_. The day before transfection, the cells were plated in 6-well plates or 150 mm culture dishes (Costar) without antibiotics at a density of 30–40 %. Transfections of all of the DNA or RNA duplexes were performed using Lipofectamine 2000 (Invitrogen) or Entranster-R (Engreen) transfection reagent according to the manufacturer’s recommendations.

### Fluorescence microscopy

Synthesized, fluorescently labeled dsRNA molecules were transfected into PC-3 cells. The cells were imaged using converted fluorescence microscopy 24 h or 48 h after transfection. Cultured cells were washed twice with PBS containing the RNase inhibitor RNasin (50 units/ml final) before each observation to reduce non-specific background interference. For densitometry analysis, the relative fluorescence intensity in the nucleus was measured using Image J software (http://rsbweb.nih.gov/ij).

### Luciferase reporter assay

PC-3 and T-24 cells were seeded in 96-well plates, and p21 promoter-pGL3 constructs were co-transfected with dsP21-322 or dsControl (50 nM). The activities of firefly luciferase and Renilla luciferase were measured 48 h post-transfection using a Dual-Luciferase Reporter Assay Kit (Promega) following the manufacturer’s protocol. Firefly luciferase activity was normalized to that of Renilla luciferase for each sample.

### Nucleic acid extraction and RNA analysis

Total cellular RNAs were isolated using TRIzol reagent (Invitrogen). Expression of the p21 and p53 promoters was evaluated using RT-qPCR. Each RNA sample was treated with RNase-free DNase I (Qiagen) to remove any potential contaminating DNA. Samples of total RNA (2 μg) were used for cDNA synthesis using SuperScript reverse transcriptase (Invitrogen) and oligo (dT) primers or random primers. qPCR was performed to quantify gene expression using Platinum SYBR Green qPCR SuperMix-UDG (Invitrogen) and the Stratagene Mx3000™ sequence detection system (Stratagene). The qPCR-specific primer sequences are listed in Additional file 1: Table S1.

### Western blotting

Cultured cells were washed twice with cold PBS containing a protease inhibitor cocktail (1 μl/ml final). Cellular lysates were prepared by incubating the cells in lysis buffer (50 mM Tris–HCl, pH 7.5, 150 mM NaCl, 0.5 % NP-40 and 2 mM EDTA) containing the protease inhibitor cocktail in DEPC-treated water for 30 min at 4 °C, which was followed by centrifugation at 14,000 *g* for 15 min at 4 °C. The protein concentration of the lysates was determined in the supernatant fraction using a BCA protein assay kit according to the manufacturer’s protocol (Pierce, Rockford, IL). For Western blot analysis, the cell lysates were resolved using 10 % SDS-PAGE gels and transferred onto nitrocellulose membranes (Millipore). Membranes were incubated with the appropriate antibodies for 1 h at room temperature or overnight at 4 °C followed by incubation with a secondary antibody. Immunoreactive bands were visualized using Luminol Reagent (Santa Cruz) according to the manufacturer’s recommendation.

### Chromatin immunoprecipitation (ChIP) assay

The ChIP assays were performed using a ChIP assay kit (17–371; Millipore) according to the manufacturer’s instructions. A total of 3.5 × 10^6^ cells was used for each immunoprecipitation. The following antibodies were used for the immunoprecipitations: anti–Biotin, anti-AGO1, anti-AGO2, anti-RNA polymerase II, anti-H3k4m3 and normal mouse IgG. A total of 5 μg of each of the appropriate antibodies was used for each ChIP. Immunoprecipitated DNA was reverse cross-linked, purified, and analyzed using qPCR. Primers used for ChIP are described in Additional file 1: Table S1.

### Statistical analysis

Results are expressed as the means ± S.D. Statistical analyses were performed using SPSS 15.0 statistical software (SPSS, Chicago, IL, USA). Student’s t-test and one-way ANOVA followed by Dunnett’s multiple comparison tests were adopted. Differences were considered statistically significant at *P* < 0.05.

## Results

### A dsRNA targets the p21 promoter and induces gene expression

**Fig. 1:**
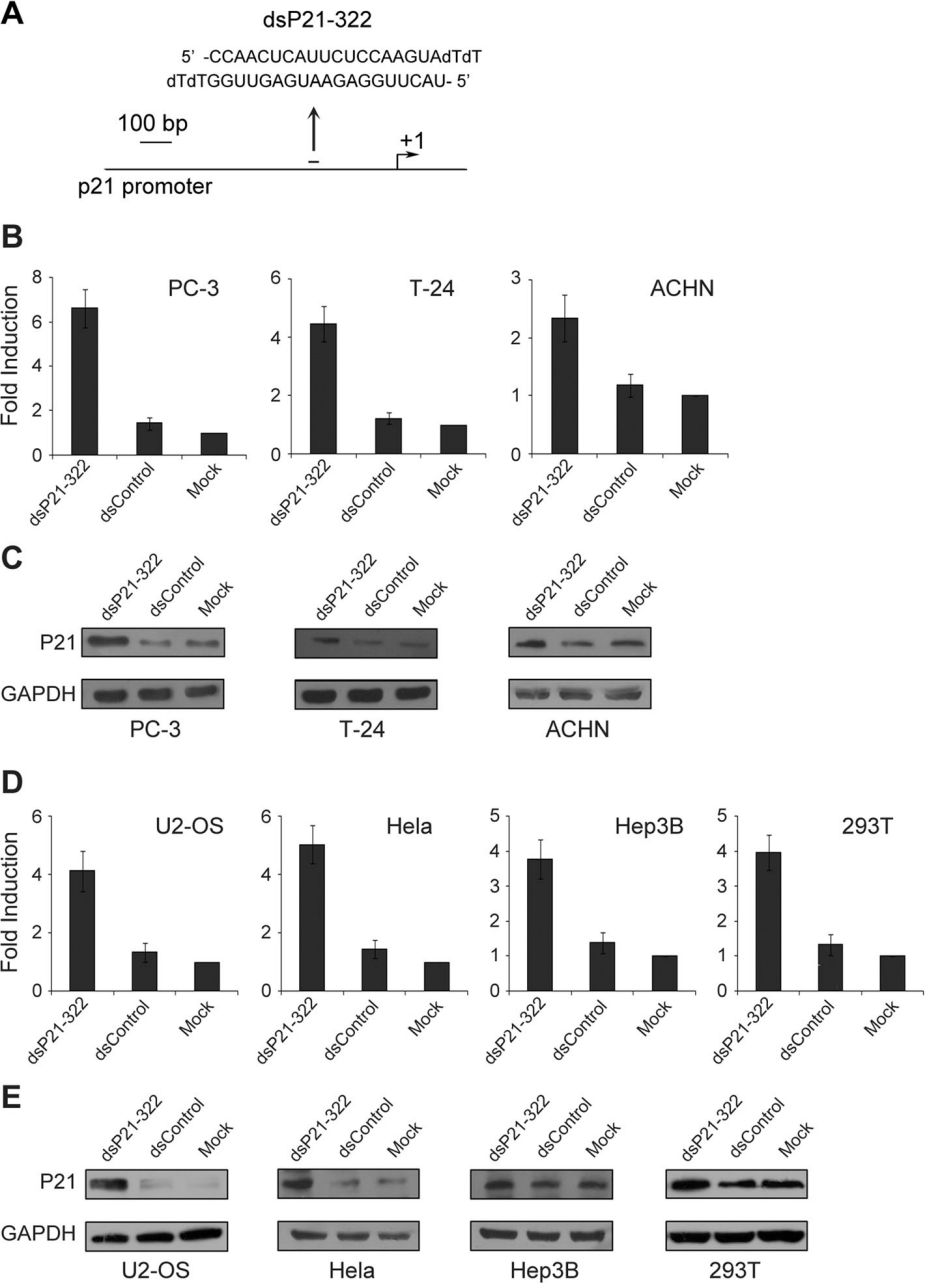
dsRNAs targeting the p21 gene promoter induce p21 expression in different human cell lines. Cells were transfected with 50 nM dsRNA for 72 h. The mRNA and protein levels were analyzed using RT-qPCR and Western blotting, respectively. **a** A schematic representation of the p21 promoter with its transcriptional start site and the dsRNA target. **b** p21 and 18S rRNA expression levels were assessed using RT-qPCR in PC-3, T-24, and ACHN cells after dsP21-322, dsControl, or mock transfections. The p21 expression level was normalized to that of 18S rRNA and plotted as fold change relative to the mock treatment. The results are expressed as the mean ± S.D. of three independent experiments. **c** The induction of p21 protein expression was confirmed by Western blot analysis in PC-3, T-24, and ACHN cells. GAPDH levels were also detected and served as a loading control. **d** p21 mRNA expression was analyzed using RT-qPCR, and the results were normalized to 18S RNA in U2-OS, HeLa, Hep3B, and 293T cells after dsP21-322, dsControl, or mock transfections. The results are expressed as the mean ± S.D. for three independent experiments. **e** Western blot analysis of p21 and GAPDH after dsP21-322, dsControl, or mock transfections in U2-OS, HeLa, Hep3B, and 293T cells

A dsRNA targeting the p21 promoter at position-322 relative to the transcription start site (dsP21-322) was used to activate p21 expression as previously described [19] (Fig. 1a). A dsRNA (dsP21-322) and a nonspecific control dsRNA (dsControl) were transfected into PC-3 (prostate adenocarcinoma) cells, T24 (bladder cancer) cells and ACHN (renal carcinoma) cells. The expression levels of p21 were evaluated 72 h or 96 h later using quantitative real-time PCR (RT-qPCR) and immunoblotting. As shown in Fig. 1b and c, dsP21-322 significantly increased p21 mRNA and protein levels compared with mock and dsControl transfections in the aforementioned urologic tumor cells. We further examined the activity of dsP21-322 in four additional human cell lines, including the human osteosarcoma cell line U2-OS, the human cervical carcinoma cell line HeLa, the human hepatoma cell line Hep3B, and the embryonic kidney cell line 293T. At 72 h, dsP21–322 transfection resulted in varying degrees of p21 induction in each cell line. Compared with mock transfections, p21 induction was 4.12-, 5.03-, 3.77- and 3.97-fold higher in U2-OS, HeLa, Hep3B and 293T cells, respectively (Fig. 1d). Induction of p21 was further confirmed using Western blot analysis (Fig. 1e), and the elevated levels of p21 protein strongly correlated with the increase in p21 mRNA in all four human cell lines. These data indicate that saRNA-mediated activation of p21 expression in human cell lines is a common phenomenon.

### Moment analysis of the kinetics of dsRNA subcellular distribution and RNAa-mediated induction of p21 gene expression

**Fig. 2:**
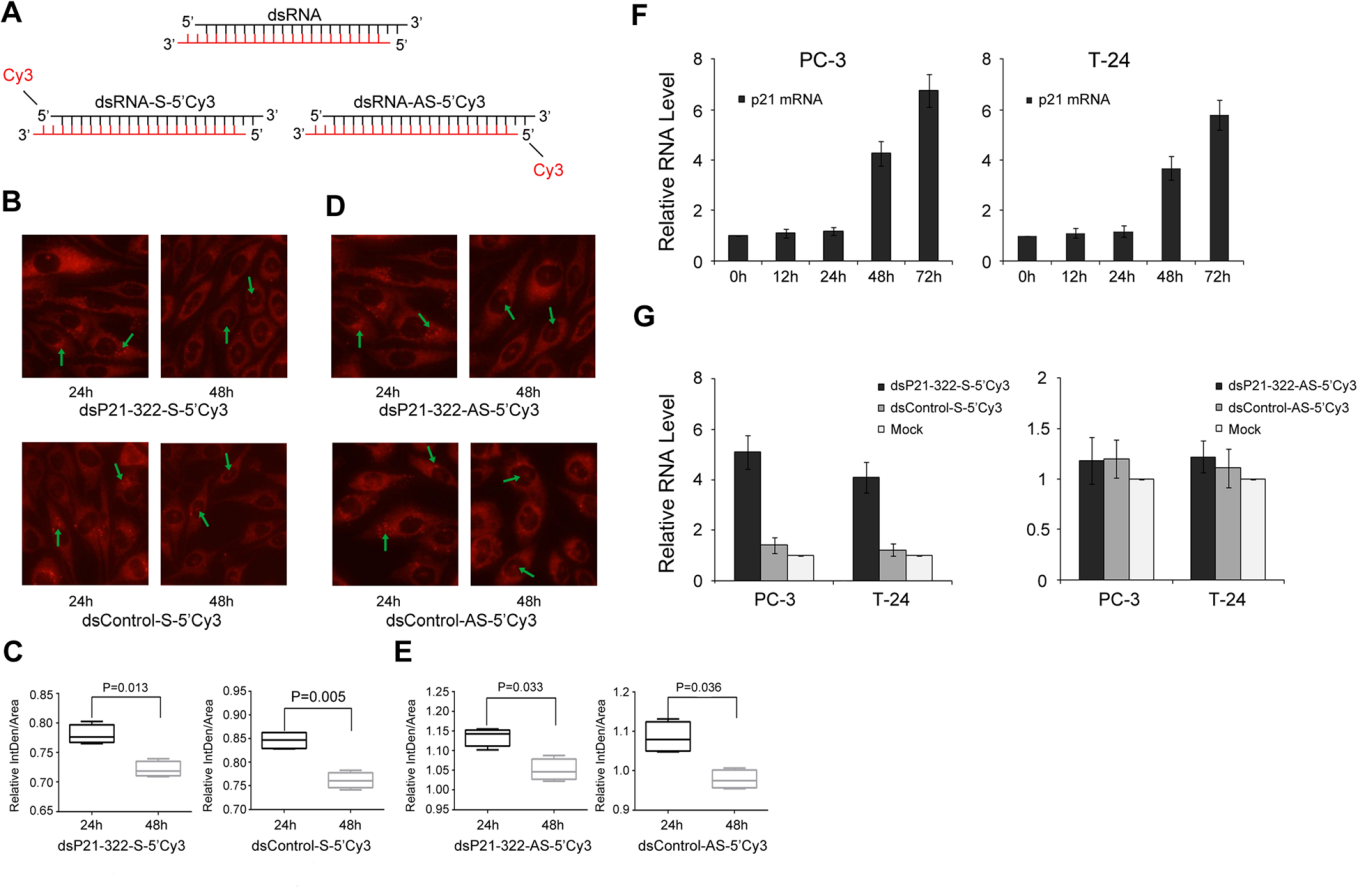
Moment analysis of the kinetics of dsRNA subcellular distribution and RNAa activity. **a** Schematic representation of modified dsRNAs (dsP21-322 or dsControl) that were covalently linked to Cy3 at the 5’-end of either the sense (dsRNA-S-5’Cy3) or antisense (dsRNA-AS-5’Cy3) sequences. The sense strand of each duplex is shown in black, and the antisense strand is shown in red. **b** PC-3 cells were transfected with 50 nM of fluorescently labeled dsRNAs for 24 h or **c** 48 h to monitor the dsRNA subcellular distribution. **d** PC-3 and T-24 cells were transfected with 50 nM dsP21-322 for the indicated lengths of time to monitor target gene induction via RNAa. Expression levels of p21 were assessed using RT-qPCR. 18S RNA was also evaluated and served as a loading control. The results are expressed as the mean ± S.D. of three independent experiments. **e** p21 and 18S rRNA expression levels were assessed using RT-qPCR in PC-3 and T-24 cells after dsRNA or mock transfections. The p21 expression level was normalized to that of 18S rRNA and plotted as the fold change relative to the mock treatment. The results are expressed as the mean ± S.D. of three independent experiments

To monitor the subcellular location of the dsRNA, we synthesized fluorescently labeled dsRNA molecules derived from dsP21-322 or dsControl that were covalently linked to Cy3 either at the 5’-end of the sense (dsP21-322-S-5’Cy3 and Control-S-5’Cy3) or the antisense (dsP21-322-AS-5’Cy3 and Control-AS-5’Cy3) sequence (Fig. 2a). Twenty-four hours after the transfection of PC-3 cells with labeled dsRNAs, the cells were imaged using converted fluorescence microscopy to observe the delivery of dsP21-322 or dsControl into the cytoplasm (Fig. 2b and d). However, most of the dsRNAs actively migrated into the nucleus from the cytoplasm 48 h after transfection (Fig. 2b and d). For densitometry analysis, the relative fluorescence intensity in the nucleus was measured using Image J software (http://rsbweb.nih.gov/ij). The smaller the ratio of the IntDen/Area, the greater the intensity of fluorescence. The results previously mentioned are showed in Fig. 2c and e. To further investigate the rate of p21 gene induction using saRNA, we transfected PC-3 or T-24 cells with dsP21-322 and monitored the cells throughout a 72 h time course. As shown in Fig. 2f, the induction of p21 gene expression in both cell lines was detectable 48 h after transfection, and the levels continued to rise until 72 h post-transfection, indicating that saRNA-mediated p21 induction occurs in the nucleus. We also found that the RNAa activity of fluorescently modified dsP21-322 (dsP21-322-S-5’Cy3) resulted in a significant induction of p21 mRNA expression, whereas dsP21-322-AS-5’Cy3 completely prevented this RNAa activity despite nuclear localization (Fig. 2g). These results demonstrated that the saRNA migrated into the nucleus and successfully entered the RNAa pathway to mediate sequence-specific activation 48 h after saRNA transfection.

### p53 is not required for dsP21-322-induced p21 expression

**Fig. 3:**
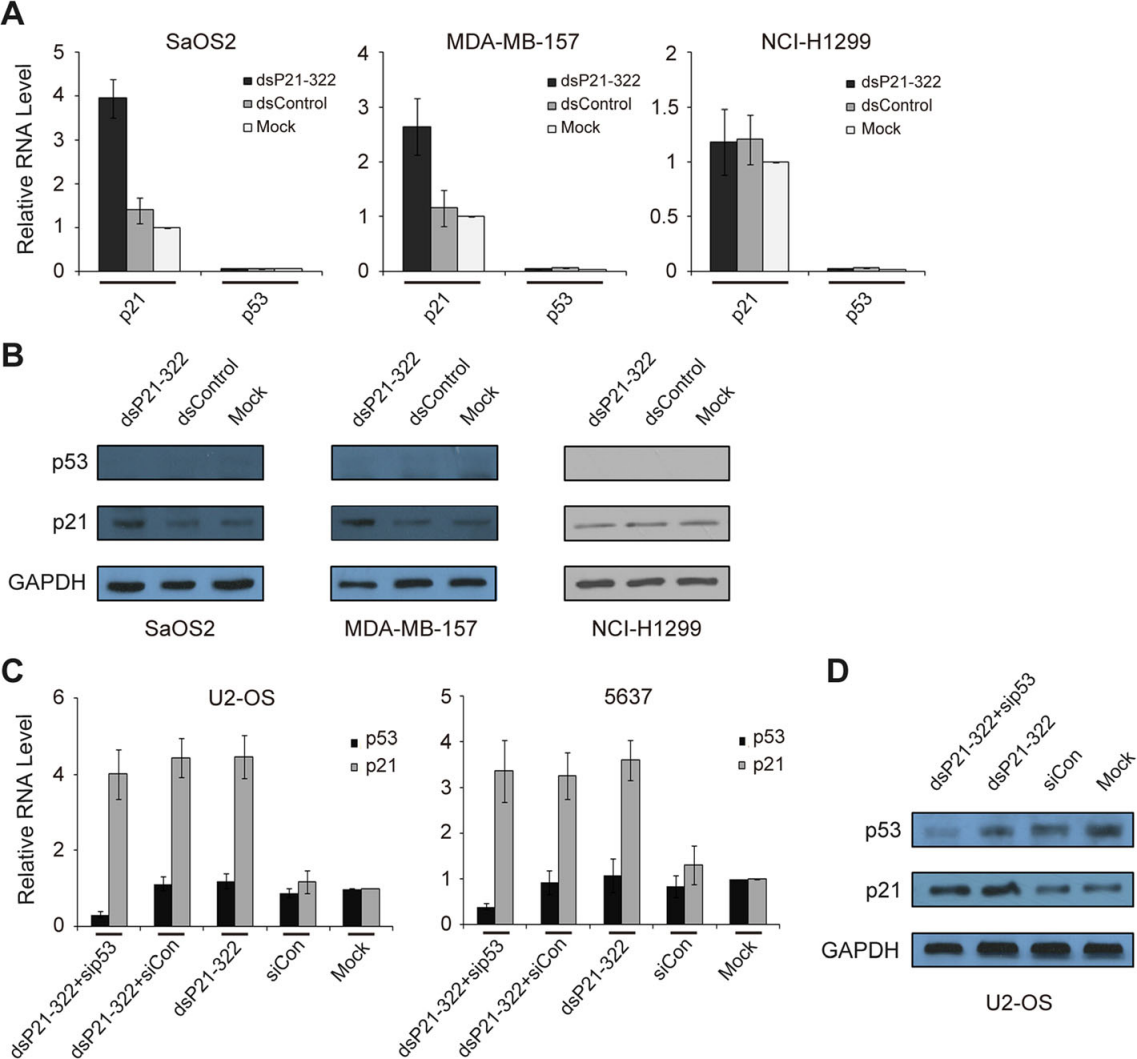
p53 is not required for dsP21-322-induced p21 expression. **a** The p21, p53 and 18S rRNA expression levels were assessed using RT-qPCR in Saos2, MDA-MB-157 and NCI-H1299 cells after dsP21-322, dsControl, or mock transfections. The p21 and p53 expression levels were normalized to that of 18S rRNA and plotted as fold change relative to the mock treatment. The results are expressed as the mean ± S.D. of three independent experiments. **b** Induction of p21 and p53 protein expression was confirmed using Western blot analysis in Saos2, MDA-MB-157 and NCI-H1299 cells. GAPDH levels were also detected and served as a loading control. **c** U2-OS and 5637 cells were transfected with 50 nM control siRNA (siCon) or dsP21-322. Combination treatments of dsP21-322 and control siRNA or p53-specific siRNA (sip53) were performed with 50 nM of each RNA duplex. Mock samples were transfected in the absence of dsRNA. Control siRNA consists of scrambled dsRNA based on the p53 siRNA. p21, p53 and 18S rRNA expression levels were assessed using standard RT–qPCR. Data were plotted as the fold change relative to the mock cells. The results are expressed as the mean ± S.D. of three independent experiments. **d** p53 and p21 protein expression levels were confirmed using immunoblot analysis in U2-OS cells. GAPDH levels were also detected and served as a loading control

To investigate whether p53, a transcriptional activator, is required for the dsP21-322-mediated activation of p21, we selected three different p53-null human cell lines, including the human osteosarcoma cell line SaOS2, the human breast cancer cell line MDA-MB-157 and the human non-small cell lung cancer cell line NCI-H1299, to examine the activity of dsP21-322. Seventy-two hours after dsP21-322 transfection, we observed a similar activation effect of the saRNA on p21 mRNA and protein expression in all of the cell lines, except for NCI-H1299 (Fig. 3a and b). We next performed a loss-of-function study for p53. We transfected U2-OS cells (p53 wild type) and the human bladder cancer cell line 5637 (p53 mutant) with siRNAs targeting p53 in combination with dsP21-322. The effect of reduced p53 expression on the activity of dsP21-322 was evaluated using RT-qPCR and immunoblotting after 72 h and 96 h, respectively. As shown in Fig. 3c, the inhibition of p53 expression did not prevent p21 induction by dsP21-322 in both cell lines. We also observed similar dsP21-322-mediated elevation of p21 protein expression after p53 knockdown in U2-OS cells (Fig. 3d), suggesting that p53 is not a component of the dsP21-322-induced p21 expression effector complex.

### dsP21-322-mediated p21 expression occurs at the transcriptional level

**Fig. 4:**
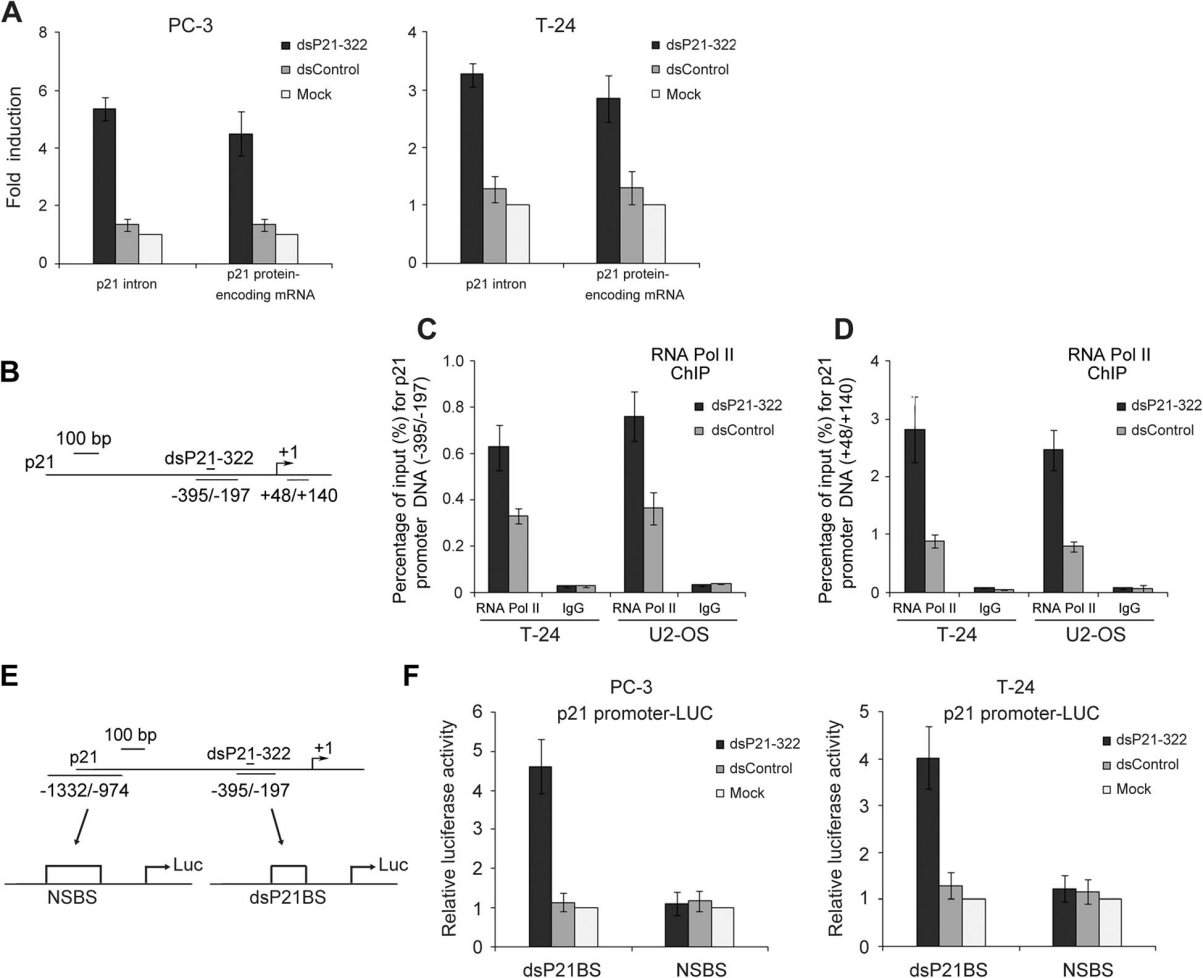
dsP21-322-directed p21 expression occurs at the transcriptional level. **a** The p21 intron, p21 protein-encoding mRNA and 18S rRNA expression levels were assessed using RT-qPCR in PC-3 and T-24 cells after dsP21-322, dsControl, or mock transfections. The p21 intron and p21 protein-encoding mRNA expression levels were normalized to that of 18S rRNA and plotted as the fold change relative to the mock treatment. **b** Schematic representation of the locations of the PCR primers that were used in ChIP to amplify the p21 promoter or transcriptional start site. **c** A ChIP assay was performed using an anti-RNA Pol II antibody to pull down dsP21-322-targeted promoters or **d** the p21 transcriptional start site associated with RNA Pol II in T-24 and U2-OS cells. The resulting DNA was amplified using qPCR and normalized to input levels. **e** Schematic representation showing the dsP21-322 binding site (dsP21BS) on the p21 promoter and the nonspecific binding site (NSBS) luciferase reporter. **f** A luciferase reporter assay for p21 in PC-3 and T-24 cells transfected with the indicated dsP21-322 and luciferase reporter genes. The input consists of nuclear extract prior to treatment with the antibody. IgG serves as the negative control antibody. The results are expressed as the mean ± S.D. calculated from triplicate independent transfection experiments with triplicate qPCR measurements for each

To determine whether dsP21-322-mediated p21 expression occurs at the transcriptional level, we designed a primer set to introduce an intron in the p21 pre-mRNA, which can be used to determine whether gene modulation occurs before or after splicing and to amplify the corresponding RNA species. As shown in Fig. 4a, the addition of dsP21-322 to PC-3 and T-24 cells increased the levels of p21 protein-encoding mRNA. We also observed increased levels of the p21 intron, suggesting that RNA modulation occurs before splicing. Moreover, the addition of dsP21-322 enhanced the association of RNA Pol II with the dsP21-322-targeted p21 promoter (Fig. 4c) and the p21 transcriptional start site (Fig. 4d) in T-24 and U2-OS cells, indicating that RNA activation occurs at the transcriptional level. Our previous studies indicated that promoter-targeted dsP21-322 can directly interact with its target promoter. To further verify this finding, the p21 promoter encoding the putative dsP21-322 binding site (dsP21BS) was cloned upstream of the luciferase open reading frame (Fig. 4e). This reporter was co-transfected into PC-3 or T-24 cells with either dsP21-322 or dsControl, and a luciferase reporter assay showed that dsP21-322 can activate the p21 promoter-luciferase reporter gene 4 to 5-fold more than the dsControl or a mock reporter (Fig. 4f). To confirm the specificity of the identified dsP21-322 binding site in the p21 promoter, we generated a p21 promoter-luciferase reporter gene with a nonspecific binding site (NSBS) (Fig. 4e), and dsP21-322 was unable to activate this reporter gene (Fig. 4f). These results are consistent with previous observations and indicate the specificity of dsP21-322 for the p21 promoter.

### dsP21-322-mediated upregulation of p21 correlates with increased AGO1 and H3K4me3 enrichment at the p21 promoter

**Fig. 5:**
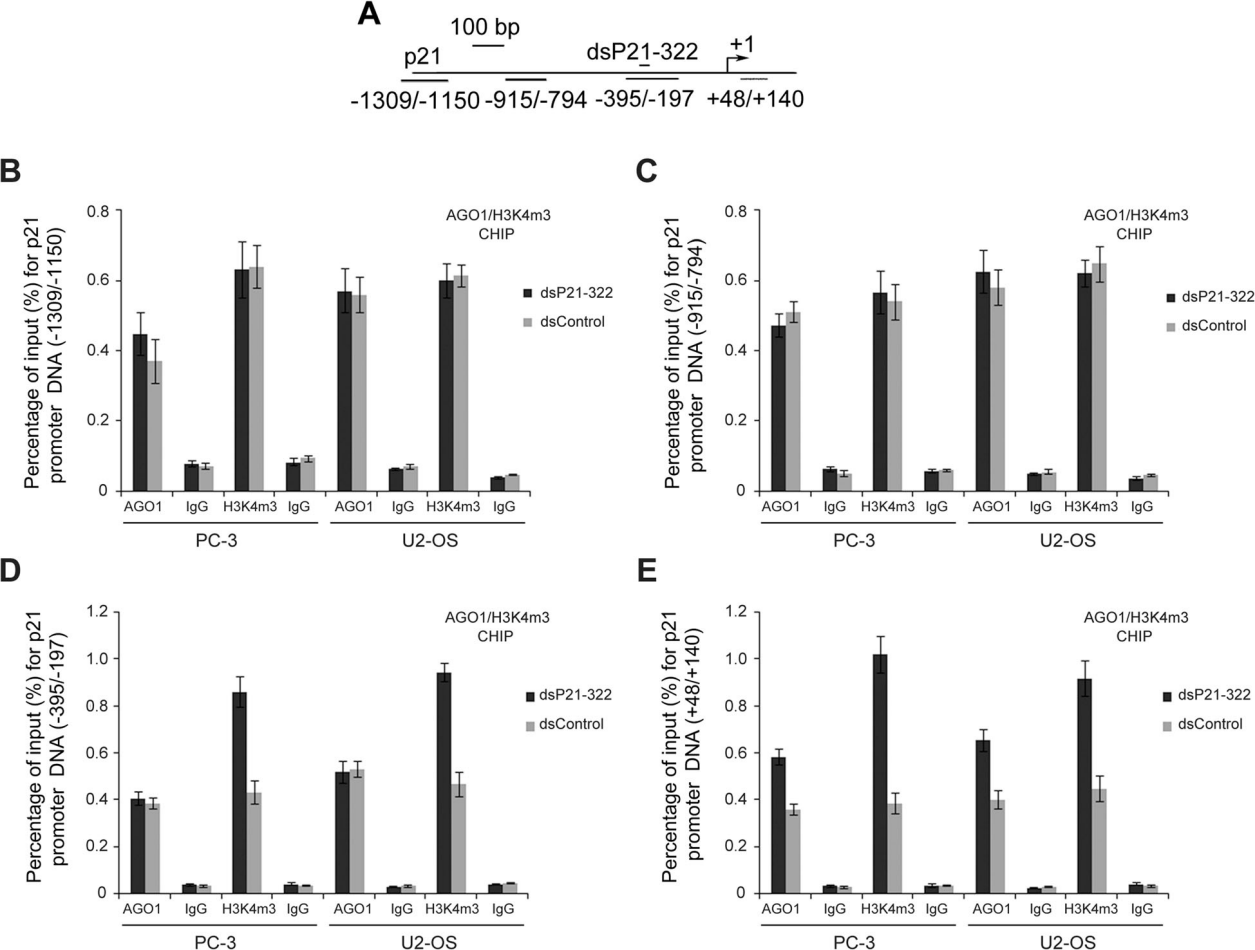
Enrichment of AGO1 and H3K4me3 at dsP21-322-targeted promoters or the p21 transcription start site (TSS). **a** Schematic representation of the locations of the PCR primers used for scanning ChIP analysis of 1.5 kb of the proximal p21 promoter. The locations are shown relative to the TSS. A ChIP assay was performed using an anti-AGO1 or anti-H3K4me3 antibody to pull down the p21 promoter upstream relative to the TSS (**b** and **c**), dsP21-322-targeted promoters **d** or p21 TSS-associated with AGO1 or H3K4me3 **e** in PC-3 and T-24 cells. The resulting DNA was amplified using qPCR and normalized to input levels. The input consists of nuclear extract prior to treatment with the antibody. IgG serves as the negative control antibody. The results are expressed as the mean ± S.D. calculated from triplicate independent transfection experiments with triplicate qPCR measurements for each

AGO1 and AGO2 are involved in several types of dsRNA- and miRNA-mediated gene regulation, as demonstrated by both loss-of-function and ChIP analyses [31–33]. Our previous study revealed that AGO2 enrichment was evident at the dsP21-322 target site after transfection of dsP21-322 using a ChIP assay in PC-3 cells. We also observed a similar recruitment of AGO2 at the p21 promoter in the T-24 and U2-OS cell lines (Additional file 2: Figure S1). In this study, we performed additional ChIP scanning to examine the association of AGO1 and a transcriptional activation chromatin marker, H3K4me3, at the p21 promoter in PC-3 and U2-OS cells after dsP21-322 transfection using specific antibodies. Four primer pairs encompassing selected regions across 1.5 kb of the p21 promoter were designed to amplify DNA that was pulled down by the corresponding proteins (Fig. 5a). We quantitatively demonstrated the enrichment of AGO1 and H3K4me3 at the selected regions using qPCR. As shown in Fig. 5b-e, compared with the dsControl, the addition of dsP21-322 increased H3K4me3 enrichment, and a slight enhancement of AGO1 recruitment at the dsP21-322 target site (Fig. 5d) and the p21 transcriptional start site (Fig. 5e) was observed in both cell lines. To further study the role of AGO1 in the process of RNAa, a dsP21-322-mutant was synthesized and transfected into PC-3 cells. The ChIP results demonstrated that mutant dsP21-322 abolished AGO1 recruitment to the dsP21-322 binding site or the TSS region (Additional file 3: Figure S2). These results indicate that AGO protein and histone modification play important roles in the activation of saRNA-mediated p21 expression.

## Discussion

Recently, RNAa has been shown to activate genes that are capable of suppressing tumor cell growth (p21, E-cadherin, p53 and NKX3.1), triggering angiogenesis (VEGF), influencing stem cell maintenance (KLF4 and OCT4) and regulating endocrine or metabolism levels (PR and LDLR) [10, 12–14, 17, 21]. RNAa appears to be a widespread phenomenon that is conserved in mammals. Moreover, new findings from a series of recent studies in mice [34] and *Caenorhabditis elegans* [35, 36] have revealed an activating role for the small RNA-Argonaute pathway and established that RNAa is an endogenous regulatory mechanism of gene expression.

Understanding the mechanism upregulating gene expression by promoter-targeted saRNAs will require the identification of the molecular targets of these saRNAs, their associated key factors, and their epigenetic influence at complementary genomic loci. Data from this study using a luciferase reporter assay revealed that saRNAs associate specifically with intended targets on the p21 promoter. In combination with the results of our previous study [19], chromatin immunoprecipitation of biotinylated sense or antisense strands of the saRNA duplex demonstrated a physical interaction with the complementary DNA of the p21 promoter, suggesting that promoter sequences are the likely targets of saRNAs. To support our finding, a report by Place et al. indicated that the concurrent induction of E-cadherin and CSDC2 by endogenous miR-373 was specific to the near-perfect complementarity of the microRNA target sites in both gene promoters [11]. Similarly, Huang V. et al. also showed that Ccnb1-activating miRNAs activate Ccnb1 expression by binding to the Ccnb1 promoter in an AGO1-dependent manner [37]. In contrast to our results, studies by Schwartz et al. and Yue et al. observed no direct interaction between saRNAs and chromatin in a PR activation model and suggested that nascent overlapping transcripts of the PR promoter likely serve as the molecular targets of saRNAs [22, 38]. Although genomic studies have revealed that both sense and antisense transcripts commonly overlap in promoters and provide a wide selection of possible targets for saRNAs [39, 40], we did not detect any non-coding transcripts overlapping with the p21 promoter [19], which suggests that the specific target site for different saRNAs may differ for different genes examined. Thus, any general mechanisms of RNAa would be difficult to establish.

The posttranscriptional gene silencing mediated by siRNAs is observable within 6 h, with levels maximally decreasing in ~24 h [41], whereas the rate of gene activation by saRNAs is typically 24–48 h [10, 42]. These kinetic differences between classical RNAi and RNAa suggest that a complex mechanism with additional rate-limiting steps may play a critical role. In our study, Figs. 2b, d and 4a show that RNA activation occurs at the transcriptional level and that this process occurs in the nucleus. Acquiring access to the nucleus may be an additional rate-limiting step. In addition, a classic histone modification marker of active transcription, H3K4me3, was recruited to the p21 promoter following induction by dsP21-322 (Fig. 5), suggesting that changes in chromatin structure further contribute to the slower kinetics of RNAa. The fact that the saRNA transfection maintained gene induction for nearly 2 weeks (~12 days) also supports this notion [42]. Other studies by Janowski [12] and Huang [37] also reported that H3K4me3 is enriched at the PR and cyclin B1 promoters following induction by their respective saRNAs. Intriguingly, the specific histone changes that occur following saRNA treatment differ for the various genes and cell types examined. For example, a reduction in H3K9 acetylation (H3acK9) was associated with saRNA induction of PR [12], but no significant changes occurred at the p21 promoter after transfection with dsP21-322 [19]. Moreover, saRNAs targeting PAWR, PR, and interleukin (IL)-24 promoters have all been shown to increase dimethylated H3K4 (H3K4me2) [12, 15, 43]. However, the enrichment of H3K4me2 was not observed at either the E-cadherin or IL-32 gene promoters after saRNA treatments [10, 43].

AGO1 and AGO2 proteins are expressed in both the cytoplasm and nucleus and are well-characterized in mammals, assisting in post-transcriptional gene silencing (PTGS) [44, 45] and transcriptional gene silencing (TGS) [46, 47]. Moreover, AGO2 is involved in several types of dsRNA-mediated gene activation [10, 12, 37], as revealed with the RNA immunoprecipitation of the AGO2 protein, which showed association with hundreds of sites in transcriptionally active regions. Additionally, AGO2 was bound to small RNAs enriched in the promoter regions of many genes [48, 49]. However, the expression of AGO1 was not necessary for efficient gene activation, and AGO1 was not recruited to the target promoter or the non-coding transcript for gene activation in previous studies [31, 42]. In our report, we showed that the induction of p21 by saRNA correlates with increased AGO1 enrichment at the p21 promoter, which suggests that AGO1 also participates in activation at the transcriptional level. This finding was also supported by a recent study that suggested that nuclear AGO1 is pervasively associated with the promoters of actively transcribed genes that are involved in growth, survival, and cell cycle progression and selectively interacts with RNA polymerase II in human cancer cells [32]. Taken together, AGO1and AGO2 proteins localize to the nucleus and interact with a number of RNA molecules and protein factors, including chromatin, RNA polymerase II and epigenetic markers to induce gene activation.

Though AGO1 could not lead apparent expression change of p21 gene in the process of RNAa, AGO1 may have something to do with the control of constitutive and alternative splicing in human cancer cells [50]. Additionally, AGO proteins participated in many biological processes, not only in classical AGO-mediated gene-silencing principles, which could affect mRNA translation and decay, but also in regulating gene transcription and repairing damaged DNA double-strands [45]. To sum up, we hypothesized that AGO1should probably have something to do with the function chromatin modification of AGO proteins to a certain extent, such as the histone methylation and acetylation. Even the modification of AGO proteins themselves, such as phosphorylation and ubiquitin [45], could occur in that process. However, there are still major gaps in our understanding of AGO1 function at the molecular level in the process of dsP21-322-mediated p21 activation. In the future, we will continue to elucidate the mechanism of AGO1 function in RNAa.

Knockdown of the p53 transcript by siRNAs did not prevent the significant up regulation of p21 transcription, revealing the specificity of p21 gene activation mediated by saRNA. As such, we demonstrated that saRNAs actively migrate into the nucleus and recruit AGO protein to complementary elements within the p21 promoter. Chromatin DNA-saRNA interactions act as scaffolds for transcription factor recruitment and trigger histone modification, which may be a general mechanism for saRNA-controlled p21 gene expression.

## Conclusions

In summary, the meager evidence currently available makes it difficult to understand the general mechanisms of RNAa, particularly the target molecules of saRNAs. Our findings distinctly revealed the saRNA-mediated mechanistic and functional aspects of tumor suppressor gene p21 activation, which may offer a novel approach for gene therapies. The ability to selectively upregulate TSG expression against a disease state can have a far-reaching impact on basic research and the development of cancer therapeutics.

## Additional files

Additional file 1: Table S1. Primers used in this paper and their targets. (DOC 29 kb) Additional file 2: Figure S1. Enrichment of AGO2 at dsP21-322-targeted promoters or the p21 TSS. (TIF 1016 kb) Additional file 3: Figure S2. dsP21-322-mutant abolished to recruit AGO1 protein to dsP21-322 binding site or the transcription start site (TSS). (TIF 1962 kb)

## Abbreviations

AGO1: Argonaute 1
AGO2: Argonaute 2
ChIP: Chromatin immunoprecipitation
hnRNPA2/B1: Heterogeneous nuclear ribonucleoproteins A2/B1
ncRNAs: Non-coding RNAs
RNAa: dsRNA-induced genes activation
saRNA: Small activating dsRNA
siRNAs: Short interfering RNAs
TSG: Tumor suppressor genes
TSS: Transcription start site
