# Supplementary material for: Demystifying the mechanistic and functional aspects of p21 gene activation with double-stranded RNAs in human cancer cells
Source: J Exp Clin Cancer Res. 2016 Sep 17;35:145. doi: 10.1186/s13046-016-0423-y (PMC5027115; doi:10.1186/s13046-016-0423-y)
Supplement: Additional file 1: Table S1. — Primers used in this paper and their targets. (DOC 29 kb) [file 13046_2016_423_MOESM1_ESM.doc]

**Additional file 1: Table S1**

Primers used in this paper and their targets.

Primers were used for either qPCR or ChIP.

**Name Sequence Assay used for Gene Targeted**

p21-1309/-1150F 5’ GAGCAGCCTGAGATGTCAGTAATT 3’ ChIP p21 promoter

p21-1309/-1150R 5’ TCCCCTGGACTTCACCTTTG 3’

p21-915/-794F 5’ TTTGCTGCATGATCTGAGTTAGG 3’ ChIP p21 promoter

p21 -915/-794R 5’ AAGGGGAGGATTTGACGAGTG 3’

p21 -395/-197F 5’ TAATGTCATCCTCCTGATCTTTTCA 3’ ChIP p21 promoter

p21 -395/-197R 5’ TCGCCTGCGTTGGTGC 3’

p21+48/+140F 5’ GCCGAAGTCAGTTCCTTGTGG 3’ ChIP p21 TSS

p21+48/+140R 5’ GTTGTCTGCCGCCGCTCT 3’

p21 intron F1 5’ CCGAAGTCAGTTCCTTGTGG 3’ qPCR p21mRNA

P21 intron R1 5’ GGTCCCCTGTTGTCTGCC 3’

p21F 5’ AGCAGCGGAACAAGGAGT 3’ qPCR p21mRNA

p21R 5’ CGTTAGTGCCAGGAAAGACA 3’

p53F1 5’ GTTCCGAGAGCTGAATGAGG 3’ qPCR p53 mRNA

p53R1 5’ TCTGAGTCAGGCCCTTCTGT 3’

18s rRNA F 5’ GTAACCCGTTGAACCCCATT 3’ qPCR 18s rRNA mRNA

18s rRNA R 5’ CCATCCAATCGGTAGTAGCG 3’
