# Supplementary figures and images for: Demystifying the mechanistic and functional aspects of p21 gene activation with double-stranded RNAs in human cancer cells
Source: J Exp Clin Cancer Res. 2016 Sep 17;35:145. doi: 10.1186/s13046-016-0423-y (PMC5027115; doi:10.1186/s13046-016-0423-y)

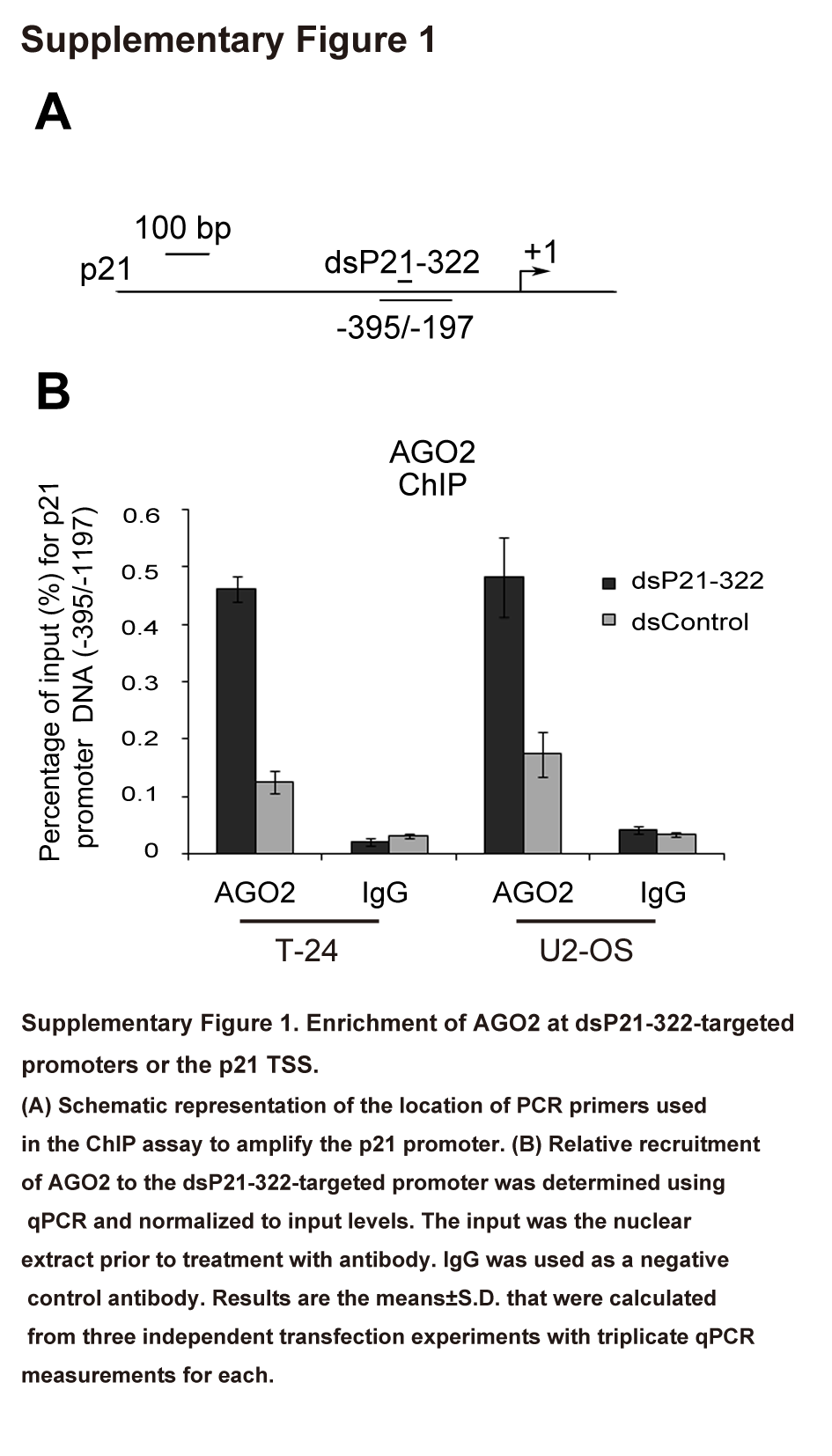

Supplement: Additional file 2: Figure S1. — Enrichment of AGO2 at dsP21-322-targeted promoters or the p21 TSS. (TIF 1016 kb) [file 13046_2016_423_MOESM2_ESM.tif]

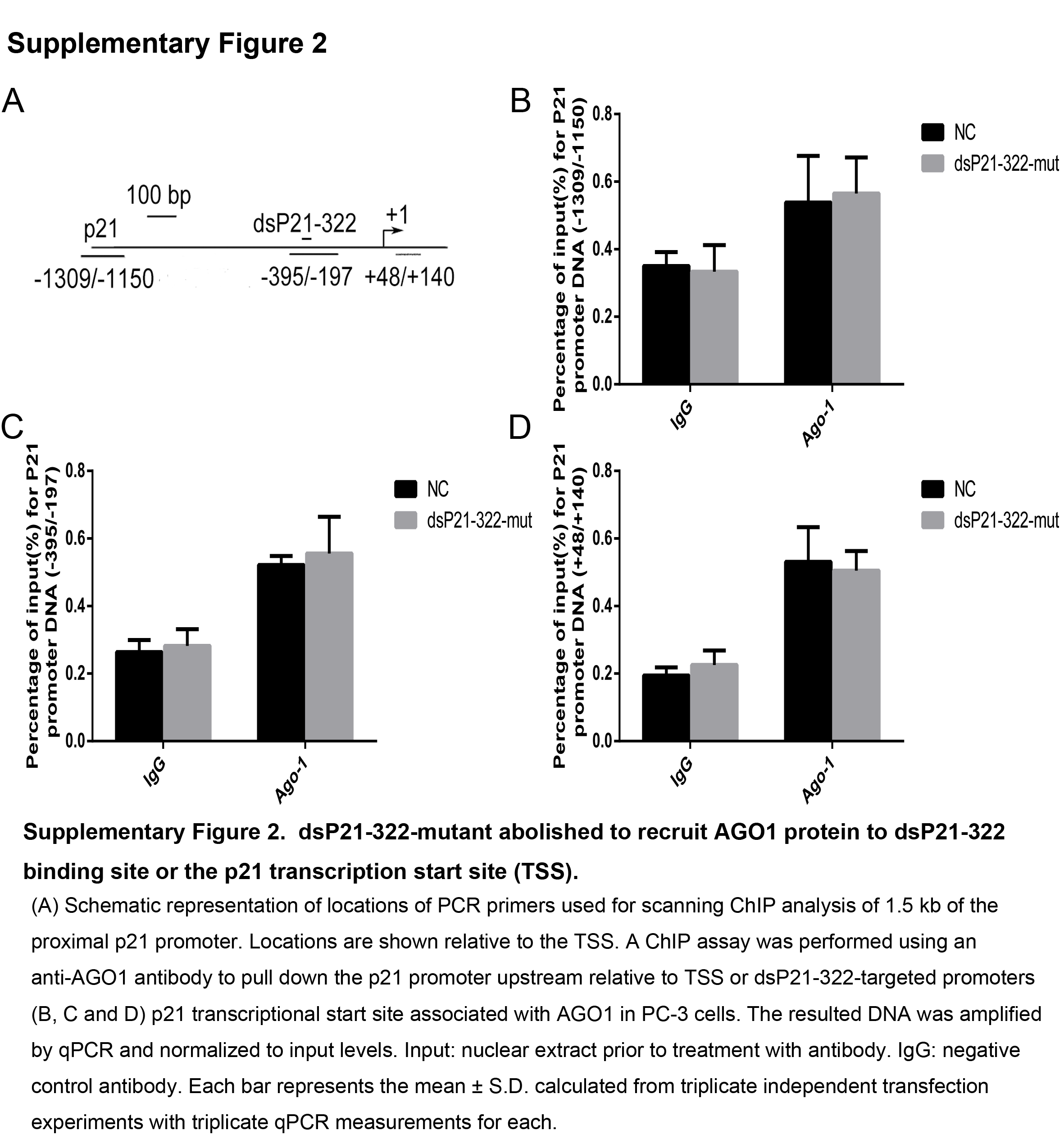

Supplement: Additional file 3: Figure S2. — dsP21-322-mutant abolished to recruit AGO1 protein to dsP21-322 binding site or the transcription start site (TSS). (TIF 1962 kb) [file 13046_2016_423_MOESM3_ESM.tif]
